# Behavioral therapy in migraine: Expanding the therapeutic arsenal

**DOI:** 10.1111/ene.16414

**Published:** 2024-07-21

**Authors:** Ane Mínguez‐Olaondo, Patricia Alves Días, Estibaliz López de Munáin, Vesselina Grozeva, Carmen Laspra‐Solís, Inés Martín Villalba, Valvanuz García‐Martín, Marta Vila‐Pueyo, Myriam Barandiarán, Ramon J. Zabalza, Ana Bengoetxea

**Affiliations:** ^1^ Neurology Department Hospital Universitario Donostia‐Osakidetza , Neuroscience Area, Biogipuzkoa Health Institute Donostia Spain; ^2^ Athenea Neuroclinics Donostia Spain; ^3^ Department of Medicine and Department of Physical Therapy, Faculty of Health Sciences University of Deusto Bilbao and San Sebastian Spain; ^4^ Psiquiatric Department Hospital Bidasoa Irún Spain; ^5^ Neurology Practice Sofia Bulgaria; ^6^ Department of Psychiatry and Clinical Psychology University Clinic of Navarra Madrid Spain; ^7^ Hospital Clínic i Provincial de Barcelona Department of Neurology Spain; ^8^ Headache and Neurological Pain Research Group, Vall d'Hebron Research Institute, Department of Medicine Universitat Autònoma de Barcelona Barcelona Spain; ^9^ Unité de Recherche en Sciences de l'Ostéopathie, Faculté des Sciences de la Motricité Université Libre de Bruxelles Brussels Belgium

**Keywords:** behavioral, disability, management, migraine, therapy

## Abstract

**Background and purpose:**

The US Headache Consortium developed evidence‐based guidelines for the treatment of migraine and found grade A evidence in support of behavior therapy (BT). Understanding the mechanisms of BT may improve the management of migraine and reduce its burden.

**Methods:**

We performed a narrative review to define the current evidence of BT and determine its usefulness in migraine management.

**Results:**

The information was obtained from 116 publications, with 56 of them retrieved through direct searches in PubMed (2011–2020) and the remainder selected by the authors to complete the content. BT might reduce migraine impact by decreasing the sympathetic nervous system's response to stress and increasing pain tolerance. Acting in headache‐related surroundings can be improved, together with headache duration and self‐efficacy. Applications such as mobile health and electronic health applications can help to carry out healthier lifestyle patterns. Regarding medication overuse, BT seems to be a good choice, with similar results to pharmacological prophylaxis. Advantages of using BT are the lack of adverse effects and the unrestricted use in children, where BT is postulated to be even more effective than the standardized pharmacopeia.

**Conclusions:**

BT is an interesting tool that can be used as an add‐on therapy in migraine. Through BT, the autonomy and empowerment of migraine patients is enhanced. BT may not cure migraine, but it could help to reduce pain severity perception, disability, and migraine impact, adding an emotive and cognitive approach to the perceptive role of pharmacopeia. Thus, a better approach in migraine, implementing specific therapeutic management, can improve migraine control.

## Graphical abstract

Behavior therapy is a useful tool with long‐lasting value. Long‐term migraine treatment should focus on adherence to therapy as well as on medical information and lifestyle modification options. Data from this study encourage the incorporation of nonpharmacological interventions in migraine treatment and the investigation of these techniques to profile the patient features for which each evaluated technique may be more suitable and thus to obtain a greater return on the investment made while working on it.

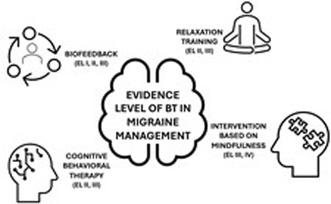

## INTRODUCTION

Migraine is a frequent and disabling neurological condition that affects 10%–16% of the general population [1, 2, 3, 4, 5], with a clear predominance in women [6]. It is the leading cause of disability between 16–50 years in developed countries, according to the most recent data obtained from global studies of diseases and risk factors [7, 8]. Recently, migraine was found to be the costliest neurological disorder [9, 10], not only because of the economic impact but also because it reduces the quality of life [11], especially for patients with the chronic form. Therefore, migraine treatment should focus on managing both the pathophysiological and psychological aspects [12] and encourage a balanced healthy lifestyle.

As for many other chronic pain situations, pharmacological treatments are the first choice even if a biopsychological conceptual approach is widely approved in medicine [13]. In recent years, increasing knowledge on pain neuronal networks has shown three key dimensions of pain experience [14]: perceptive, emotive, and cognitive. Medical treatment usually focuses on the perceptive dimension, whereas an increasing number of studies have produced interesting results regarding efficiency, for patients' quality of life, when treatments also focus on emotive and cognitive pain experience dimensions [13, 15, 16, 17].

Behavioral therapy (BT) seems to be a good choice in migraine management, because it is used in a multidisciplinary treatment program. It appears to be an effective approach, empowering the patient through awareness of the disease. Results show that when adopting BT in migraine, an improvement ranging from 33% to 55% occurs [18]. Recent studies show that mindfulness (MF) also improved other aspects such as quality of life and self‐efficacy [19]. Even though there are not always statistically significant results, patients increase their sense of efficacy and control through the self‐management of migraine [20]. This could be the reason why adherence to nonpharmacological and BT recommendations has been associated with a better outcome [21].

Current BT options in migraine include those mentioned in Table 1 [22].

**TABLE 1:** Current BT options in migraine.

BT option	Definition
BF	A technique used to manipulate physiological responses such as heart rate through the online feedback of one's involuntary body functions, allowing the development of awareness, self‐regulation processes, and control of various physiological parameters [23]
RT	A technique that works by decreasing sympathetic activation and central pain processing [24]
CBT	A psychotherapeutic approach based on the premise that our thoughts influence how we feel and behave [25]
Interventions based on MF	A type of meditation based on nonjudgmental moment‐to‐moment awareness of one's internal and external stimuli including thoughts and feelings [26]
ACT	A technique that integrates both cognitive and behavioral therapy and MF to improve the psychological inflexibility caused by avoidance behavior and cognitive fusion that supports these behaviors

*Note*: There are also alternative delivery methods of established therapies via mobile health and electronic health applications.

Abbreviations: BF, biofeedback; BT, behavioral therapy; CBT, cognitive behavior therapy; MF, mindfulness; RT, relaxation training.

Nowadays, it is preferred to name complementary or integrative health treatments [27], instead of alternative methods. The use of BT for migraine, often used as an adjunct to pharmacotherapy, avoids the adverse effects and potential contraindications of the drugs currently prescribed, such as medication overuse, bradykinesia, and mood or weight changes, among others. Moreover, BT can be conducted by health professionals upon adequate training, and its use is especially relevant for low‐income communities [28]. It could also be a choice in particular situations when the pharmacologic choices are restricted, such as during pregnancy or due to low efficacy or lack of tolerability of pharmacologic therapies [29]. Children could also be another group of interest for BT, even as a first‐line treatment [30].

Several studies have analyzed the potential benefit of different behavioral techniques in migraine treatment or prophylaxis. Contingent negative variation is a method for examining cortical information by processing the application of event‐related potentials. See Table 2 for further information.

**TABLE 2:** Note about CNV in migraine.

CNV	*Definition*: A slow cortical event‐related potential that develops between two related stimuli *First stimulus*: Serves as a warning stimulus *Second stimulus*: Imperative stimulus requiring a motor answer by the subject Patients with migraine exhibit higher CNV amplitudes [31]. Migraine is associated with abnormal central information processing and deficient regulation of cortical excitability, as it is related to increased amplitudes and reduced habituation of the CNV. Several days before migraine attack, these abnormalities are more pronounced, demonstrating increased susceptibility of the brain to migraine‐provoking and precipitating factors [32]. *Management:* Applying prophylactic treatments, such as betablockers, antiepileptics but also nonpharmacological treatment options like BT or physical exercise, CNV parameters tend to normalize, which is interpreted as a possible equalization of the cortical disbalance, demonstrating the efficacy in migraine prophylaxis [31]. Thus, changes in this habituation are closely related to improvement in the clinical course of migraine [32].

Abbreviations: BT, behavioral therapy; CNV, contingent negative variation.

Therefore, lifestyle modifications and migraine trigger identification should be discussed with migraine patients. Currently, limited published data support the finding that lifestyle modifications and avoidance of triggers are effective in reducing migraine burden. It is usually recommended to be added to the pharmacological treatment due to the small risk for the patient [33]. However, some authors conclude that incorporating MF training into usual treatment improves short‐term efficacy [34]. There is an unmet need to implement widely for migraine care available feasible nonpharmacological approaches [35].

The aim of this article is to review the knowledge obtained from 2011 to 2020 on BT in the treatment, attack management, and prevention of migraine. As we know, promoting lifestyle changes and managing migraine symptoms with a better understanding of these therapies could possibly help us to discover new therapeutic paths or even future treatment targets. In this sense, the present review will address the different types of BT, together with the key factors to take into consideration to identify the most suitable patients for this approach. With the aim of identifying the most suitable patients, we will analyze three main factors: patient profile, burden of migraine, and comorbidities.

## METHODOLOGY

A search of PubMed and Web of Science databases was used to identify publications from 2011 to 2020 related to behavior therapies and migraine. The search strategy included the Medical Subject Headings key terms “behavior therapy AND migraine” and “behavior therapy AND migraine disorders.” Three of the authors (A.M.‐O., P.A.D., E.L.d.M.) independently examined all the titles and abstracts retrieved and selected the articles that met the inclusion criteria. Duplicate publications were removed by manual check.

**FIGURE 1:**
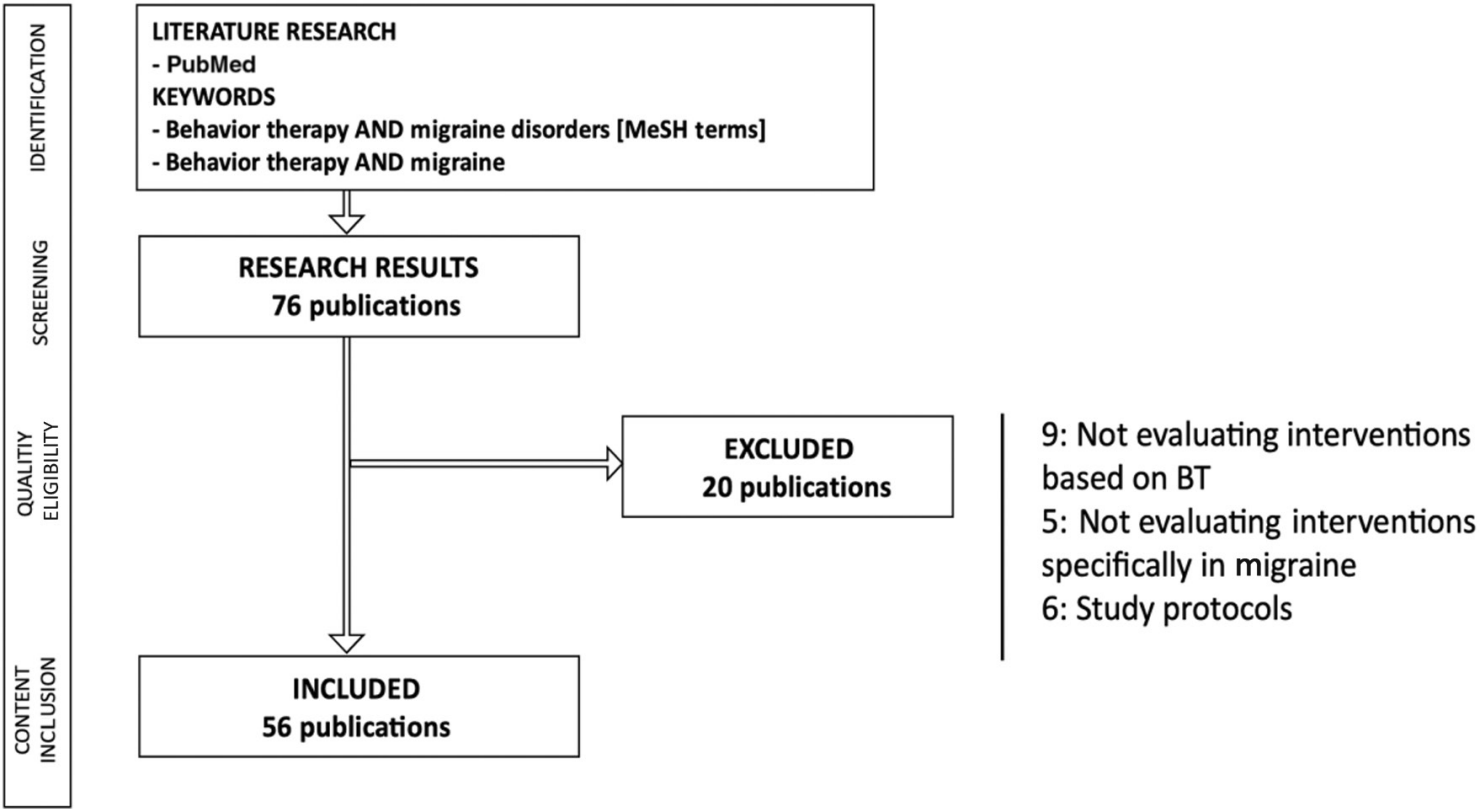
Study flow diagram. BT, behavior therapy; MeSH, Medical Subject Headings.

Studies eligible for inclusion were all types of articles published in English, exploring well‐defined BT in humans, and linked to migraine. Studies lacking a clear description of the diagnostic criteria for migraine or the type of BT were excluded. Studies published in languages other than English, study protocols, and publications not specifically mentioning migraine or not focusing on BT in migraine were excluded (Figure 1). The results were screened for relevance to the review topic. Articles up to 2023 were also added based on the authors' knowledge of the area. The criteria for each level of evidence (EL) are based on the Manual of Clinical Practice in Headaches, Diagnostic‐Therapeutic Recommendations of the Spanish Society of Neurology 2020, and the American Academy of Neurology, detailed in Annex I [36].

## TYPE OF BEHAVIORAL THERAPIES

Several BTs have been shown to be significantly effective and to have a gradual, but durable, effect for migraine treatment [37]. Behavioral self‐management with a proactive focus on attack prevention improves the outcomes of the pharmacological treatment of frequent episodic migraine [37]. Thus, patients can be actively involved, and real‐time prospective monitoring of migraine and medication use can be controlled to have real migraine awareness [38].

The US Headache Consortium developed evidence‐based guidelines for the treatment of migraine and found grade A evidence supporting BT [39]. However, several studies have shown diverse results. As an example, although one of the studies found that exercise, relaxation, and topiramate intake seem to have equal effects on the reduction of migraine frequency [40], other studies have proven that the combination of pharmacological acute treatment and behavioral migraine management decrease the frequency of migraine attacks more than acute treatment alone [41, 42].

What is noted from the conclusions of these studies is that the effect of BT may take longer to set in compared to those obtained with medications, but at the same time it may be more durable. Hence, the evidence suggests that the addition of BT can improve the quality of life of patients with migraine in the long term [41]. As a result, no treatment alone is completely effective; combining different approaches seems to be the most efficient. In the case of BT, it takes more time to be efficient compared to medication but also remains efficient longer. The next sections will review the studies according to the type of BT used (Table 3).

**TABLE 3:** Selection approach depending on individual features, under a set of circumstances (scheme based on the text).

	Patients profile	Burden‐related effects	Advantages	Disadvantage	Notes
BF	Patients with stressful conditions	Improves anxiety, depression	Long‐term efficacy	The need of an instrument Costly and time‐consuming	Better outcome combined with pharmacotherapy
RT	Childhood Patients with stressful conditions	Decrease in stress, pain severity, migraine‐related disability	Increase in calmness Decrease in medication consumption	The need of a coach Time‐consuming	Combining with CBT improves the efficacy Effectiveness of workplace relaxation programs
CBT	Highly motivated patients with high crisis frequency and medication overuse	Improves stress and medication use, reduces pain catastrophizing	Adaptable for independent at‐home use Long‐term benefit	Variable evidence in reducing headache frequency Deeply detailed diaries looking for triggers	Combining with pharmacology or other BT shows better outcomes than using alone
Interventions based on MF	Patients with an affected QoL and disability, self‐efficacy and psychological symptoms over headache frequency	Improves stress, anxiety, depression, pain, and patient well‐being	As useful as drugs in chronic migraine with medication overuse	Does not directly improve frequency of migraine attacks	Good option combined with conventional medical therapy

Abbreviations: BT, behavioral therapy; CBT, cognitive behavior therapy; MF, mindfulness; QoL, quality of life; RT, relaxation training.

### Biofeedback

Biofeedback (BF) is the technique of making unconscious or involuntary body processes perceptible to the senses to control them by consciousness [43]. In migraine, the aim of this method is to improve aspects of health performance and health status to reduce the impact of headache [44]. From pretreatment to posttreatment, BF therapy resulted in a decrease in duration of headache in migraine patients measured by the computerized ecological momentary assessment (EL III) [45]. However, although a reduction of severity has also been seen, the most consistent result obtained by BF is the reduction in headache frequency (21%–67%) combined with pharmacotherapy. This shows how useful BF is in both the management and prevention of migraine attacks (EL II) [22].

Nowadays, there is a wide range of BF interventions available for headache disorders, with the most traditional ones being peripheral skin temperature biofeedback, blood–volume–pulse, and electromyographic feedback, as reviewed elsewhere (EL I) [46]. However, it must be mentioned that 16 years have passed since the publication of this review, and, besides the positive outcomes of its meta‐analysis, BF has not been included as a treatment for headache disorders in clinical practice guides, probably because such interventions are not easy to incorporate into standard clinical practice, due to the additional training, engagement, and time consumption required. Therefore, level I evidence should be approached with caution.

However, in recent years, several types of BF that require more specialized training have been developed. One of them is the quantitative electroencephalogram procedure, based on neurofeedback, which appears to significantly reduce migraine frequency in patients with recurrent migraine headache (EL III) [47]. Another type of BF that is based on the control of the reflex response and habituation to painful trigeminal stimulation could be also useful in migraine. Although this technique improves migraine frequency, cutaneous allodynia—the clinical correlate of central sensitization that shows migraine is under chronification process—is not improved (EL II) [48].

Although BF training is costly and time‐consuming [44], evidence has shown an improvement not only in headache features but also in burden, including anxiety, depression, and self‐efficacy, enduring over time. These results have led the US Headache Consortium to support BF therapy efficacity [22, 39].

### Relaxation training

Relaxation training (RT) consists of a systematic procedure for achieving a physical state of relaxation and subsequent mental quietness [49]. In migraine, headaches could be exacerbated by the body's response to stress, so these relaxation techniques are based on relaxing muscle tension but also decreasing the sympathetic nervous system's response to stress [22].

There are several types of RT, such as autogenic training, diaphragmatic breathing, guided imagery, meditation, hypnosis, and progressive muscle RT [27]. The most traditionally taught RT is progressive muscle relaxation training, which reduces migraine frequency and duration by approximately 41% and 43%, respectively (EL II) [31].

Each RT approach has its own peculiarities. When a combination of trainings is implemented, that is, when adding other therapies to RT, such as cognitive behavior therapy (CBT), a positive result can be observed (EL III) [50]. Also, in workplace relaxation exercise programs, the reduction in both muscle tenderness and head/neck/shoulder pain was accompanied by a significant decrease in drug intake and lost time at work (EL II) [51]. These data altogether emphasize the remarkable cost‐efficiency aspect of this type of intervention, which yields a relevant benefit also in economic terms, with a relatively low monetary investment [51].

### Interventions based on MF

MF meditation is an evidence‐based mind–body intervention that teaches principles of introspection and its application on daily life situations, helping patients to handle illness and stress [35].

Meditation techniques might be based on spiritual or nonspiritual meditation approaches. It has been seen that spiritual meditation may lead to a better migraine pain tolerance, as the experienced headaches, although perceived as severe, seem less likely to be deemed by the participants to require pain medication. Thus, it could influence the pain experience and analgesic use, reducing the danger of medication overuse headache and health care costs [52]. Controversial results have been obtained, however, because there are also studies supporting that the spiritual content added to this training is not related to a deeper benefit regarding pain‐related features [53]. Thus, additional research is needed to examine migraine‐specific outcome measures.

MF is considered a basic human capacity to attend to sensory experiences, events, perceptions, cognitions, and/or emotions [35], where it is recommended to observe physical sensations, even painful ones, with a curious and nonjudgmental attitude [54]. MF meditation reduces pain through several brain mechanisms and various pathways such as the changing of attention mechanisms. The major distinguishing feature of all MF‐based management is accepting one's present state and not attempting to directly alter or confront aspects related to headache [55].

Current research suggests that associations between headache conditions and imbalances in the autonomic nervous system (ANS) are due to stress‐related dysregulation in the activity of the parasympathetic–sympathetic branches. In this way, MF meditation has demonstrated effectiveness in reducing pain‐related distress and in enhancing heart rate variability, a vagally mediated marker of ANS balance [56].

Meditation and relaxation provide meaningful and active management of the headaches that is more successful in relieving pain compared to other techniques such as external secular meditation [57]. As already mentioned, MF reduces the reactivity to distressing thoughts and feelings that accompany pain perception and strengthen the pain; it does not directly improve the frequency of migraine attacks, but improves psychological symptoms and increases parasympathetic activity, which can promote deep muscle relaxation that may reduce pain [29]. That is why it is postulated that MF‐based interventions are a promising option for migraine patients' well‐being at the emotional and cognitive level (EL IV) [35].

MF could be a helpful treatment tool, with considerable impact on quality of life and disability, self‐efficacy, and psychological symptoms [35]. The efficacy of MF‐based interventions has been shown, as they alleviate the suffering in migraine patients and improve their quality of life. However, this does not imply their effectiveness in reducing the number of migraine days per month [13, 15, 16, 17]. This lack of effect on reducing migraine frequency could be the reason it seems to be more effective in patients with episodic rather than chronic migraine [29].

MF interventions have been proven more beneficial than other treatment strategies. In a study comparing this intervention with an education and relaxation program consisting of standard elements taught for the study, the MF‐based stress reduction intervention group showed greater improvements in variables of psychological symptoms, pain self‐efficacy, and sensory pain perception (EL III) [34, 58]. Moreover, recent studies comparing MF to CBT show that MF has even greater effects on psychological distress [59]. Strengthening reframing negative situations and self‐regulation skills, MF‐based stress reduction intervention had a beneficial effect on headache duration, disability, and self‐efficacy (EL III) [60, 61]. Thus, the integrating of MF‐based stress reduction treatment with conventional medical therapy in the treatment protocol for patients with headache could be advised [61].

### Acceptance and commitment therapy

Acceptance and commitment therapy (ACT) is a type of behavior therapy based on six core principles: acceptance, cognitive diffusion, contact with the present, self‐in‐context, values, and committed action [62]. It has been considered the newest member of the family of MF‐based interventions [63].

On one hand, advice to avoid all headache triggers was associated with small decreases in headaches and medication consumption, being more useful for tension‐type headache than migraine. On the other hand, combining learning to cope with triggers with other treatment techniques should lead to enhanced treatment efficacy even if it is not specific to migraine [64, 65]. As an example, in a study with a 1‐day behavioral intervention workshop in patients with depression and migraine, participants assigned to the ACT plus educational workshop exhibited significant improvements in headache frequency, headache severity, medication use, and headache‐related disability (EL III) [66].

Preliminary data from a high‐frequency episodic migraine without aura group shows that ACT seems suitable because patients can learn to manage stressful conditions related to the pain onset and they become more mindful about the pain and its origin. They also increase their self‐confidence in managing pain by reducing the intake of medication, thus avoiding the onset of medication overuse and chronification of migraine [67]. In terms of the association of changes in pain acceptance and headache‐related disability, improvements in pain acceptance following two different treatments have been associated with greater reductions in headache‐related disability, suggesting a potential new target for intervention development [68].

### Cognitive behavioral therapy

CBT is based on cognitive and behavioral aspects of migraine, teaching ways to manage mental health‐related factors associated with migraine such as anxiety, depression, sleep disorders, and pain, among others [69, 70]. It requires developing skills to identify possible migraine triggers and burden [22]. With this therapy, an important clinical impact at the period after CBT implementation has been achieved, with a high rate of subjective improvement of pain and better headache coping (EL II) [71]. Patients with high frequency as well as patients at risk of medication overuse could benefit from this therapeutic management, especially when combined with bibliotherapy, which means using selected reading materials as therapeutic adjuvants (EL III) [72].

Notice that the benefit is still there in the long term [72], as it has been shown that CBT improves pain‐coping strategies and decreases headache intensity, frequency, and catastrophizing, altering pain‐related cognition in headache patients over a period of at least 1 year (EL II) [73]. This result could be very important, as cognitive factors have an influence on the severity of pain and on the interference in patients' daily activities [74]. BT also improves insomnia, as quantified by both actigraphy (by recording and specially measuring the amount and quality of sleep) and self‐report, producing changes in headache and baseline depression scores (EL III) [75]. Thus, CBT is a useful tool within a routine clinical setting and can improve medical care [73].

### Alternative delivery methods of established therapies via mobile health and electronic health applications

Migraine is an ideal disease to test smartphone‐based mind–body interventions, because of its high prevalence, cost, and disability. Some studies are highlighted in Table 4 as evidence of this and substantiate its influence on migraine pathology.

**TABLE 4:** How alternative delivery methods of established therapies via mobile health and electronic health applications affect patients with migraine.

Article	Summary of the most pertinent content
Hedborg & Muhr	A significantly higher rate of improvement in terms of headache frequency among participants in Internet‐based multimodal behavior treatment was seen, even if no differences in depression rates was found (EL II) [76].
Sorbi et al.	These online approaches improved migraine self‐management and decreased triptan use, also improving the cost‐effectiveness of the treatment, even though a clear decrease of migraine attack frequency was not observed (EL II) [77].
Minen et al.	As mentioned in this article, knowing it requires an engaging electronic diary, in the preliminary small study of one of these apps, participants reported that progressive muscle relaxation maintained their interest and attention (75%) and improved their stress and mood (75%) (EL II) [78].

*Note:* For this therapeutic management, support and supervision remain necessary, but the contribution of clinical psychologists can be tailored to far below the dose of regular individual psychotherapy [38]. It is postulated that this benefit could be because of the awareness of, and adjustments to, healthier lifestyle patterns. Thus, using electronic health applications for behavioral therapy of migraine could be a good way to improve migraine impact in patients [76]. Moreover, it leads to decrease overall drug consumption because of its higher efficiency [79].

Abbreviation: EL, level of evidence.

## PATIENT PROFILES

People with migraine may vary regarding the factors they believe to have an impact on their migraine attacks. Understanding control beliefs remains important and should continue to be examined within the context of contemporary notions of how self‐care impacts pain, types of treatment recommendations, and the way health professionals communicate the role that patients play in their own migraine control [80].

Patients who have high levels of disability, more than those with high headache frequency, are those who perceive the most potential benefit of BT [81]. However, when modifiable barriers to treatment are systematically challenged, such as depression, anxiety, excessive use of caffeine, sleep disorders, obesity, or medication overuse [82, 83, 84, 85, 86, 87], even patients who could be seen as relatively poor candidates for psychological treatment can improve their situation. Problematic beliefs need to be effectively challenged prior to teaching behavioral migraine handle skills. A behavioral migraine management protocol exists to improve our knowledge in this area [88].

Regarding patients' profiles in migraine, special attention is needed for patients with medication overuse and children and adolescents, where we must be even more cautious when introducing a medication.

### Patients with or at risk of medication overuse

Chronic intake of drugs for migraine attacks can lead to an increased frequency of headache, facilitating the progression of migraine [69, 82, 89]. This occurs because long‐term exposure to analgesics can alter pain modulation mechanisms [90, 91], resulting in chronic headache [55]. It is possible that overuse of analgesics is a consequence rather than the cause of migraine progression [89]. In terms of prophylactic treatment, MF‐based cognitive therapy training (MF‐CBT) seems to be a good option after withdrawal from medication overuse in patients with chronic migraine (EL II) [92].

Similar effect was found when MF‐CBT was compared with pharmacological prophylaxis but without adverse effects. Both interventions uncombined revealed significant decrease in number of monthly headache days, monthly consumption of medication for acute headache management, disability, and depressive symptoms (EL III) [92]. In blood samples, a variation in catecholamine levels has been seen in patients with chronic migraine and medication overuse headache after MF‐CBT training, similar to pharmacological prophylaxis [93]. Another option mentioned in the literature is the use of frontal electromyographic biofeedback in combination with pharmacotherapy, which could stop or at least reduce analgesic drug intake, especially in the treatment of medication overuse headache (EL III) [94].

### Childhood

BT has been shown to be useful at any age and life phase, especially in children and adolescents [30]. However, data on comparative effectiveness of several mind–body techniques in children are lacking. The psychotherapy BT program, even though a brief one, seems to be more effective than usual care for patients with idiopathic headache [95]. Several trials have been tried in this population.

When analyzing children and adolescents with chronic migraine, the use of CBT plus amitriptyline, for example, improved headache frequency and migraine‐related disability compared to only headache education and amitriptyline (EL II) [96, 97]. It works by modifying behavioral responses to pain and using active coping skills, such as RT and cognitive strategies [98, 99]. Thereby, there are specific programs such as the Headstrong Program showing lower pain severity posttreatment and less migraine‐related disability, reported by children and parents [100].

Mind–body techniques with progressive muscle relaxation exercises were also studied, with a reduction of >50% in headache days in 50% of the evaluated children; however, no large differences were found compared to transcendental meditation and hypnotherapy (EL II) [101]. In addition, music therapy in the treatment of adolescents with frequent tension type and migraine headache could be an option, although recent studies showed that it is not superior to placebo (EL III) [102]. Future studies should examine the effects of meditation in children with primary headaches.

## COMORBIDITIES

### Anxiety and depression

Migraine was associated with poorer long‐term improvement when anxiety symptoms were present [103]. However, both migraine‐related depression and stress decrease when using self‐management tools and positive symptom management strategies (EL III) [104]. Headache frequency and self‐efficacy in managing episodes improved significantly after using BT, as well as the quality of life as a secondary outcome (EL II) [105]. It has been described that patients with a comorbid mood or anxiety disorder diagnosis using BTs had larger reductions in migraine days and Migraine Specific Quality of Life Questionnaire and Headache Disability Inventory results than did participants with neither diagnosis (EL II) [106].

Although it is not specific for migraine but applies to several headaches, comparing the CBT and routine primary care showed a significant improvement in the treatment group in terms of headaches, depression, anxiety, and quality of life, maintained over long‐term periods (EL III) [107]. As shown in Table 1, despite the need for future well‐designed studies, integrated headache care is related to a decrease in anxiety and depression (EL I) [108].

### Insomnia

Insomnia affects the majority of those who seek treatment for migraine [109]. Treatment using stimulus control and sleep restriction appears promising for reducing comorbid migraine. Behavioral models of the migraine–insomnia relationship articulate how headache coping behaviors can worsen insomnia through the contribution of variable sleep/wake patterns, circadian rhythm disruption, and conditioning of physiological arousal to bedroom environment [109].

Hybrid CBT intervention for adolescents with co‐occurring migraine and insomnia has been proposed, which simultaneously targets headache and insomnia [110]. BT of comorbid insomnia in individuals with chronic migraine reduces insomnia and migraine attack frequency (EL III) [75]. As an example, when CBT for patients with insomnia was used, both headache frequency and sleep parameters improved after treatment, suggesting long‐term beneficial effects (EL III) [75]. Therefore, it is postulated that CBT for comorbid insomnia may be effective in reducing the frequency of headache among individuals with chronic migraine (EL III) [109].

### Obesity

According to the World Health Organization, obesity is defined as abnormal or excessive fat accumulation higher than what is considered healthy [111]. Significant improvements in both adiposity and headache data were observed in adolescents with obesity with migraine who participated in an interdisciplinary intervention program for weight loss that included dietary education, specific physical training, and BT. Thus, a decrease in weight, body mass index, waist circumference, headache frequency and intensity, use of acute medications, and disability was observed [112].

Behavioral intervention for reduction of body weight decreased migraine headache frequency in women with comorbid overweight or obesity (EL II) [113]; however, there are still studies in which these changes in migraine headache frequency were not significantly associated with behavioral weight loss and a migraine headache education control intervention (EL III) [63]. What is already proven is that greater weight loss as occurs in obesity surgery is related to superior migraine improvement (EL II) [114]. It would be interesting to investigate additional mechanisms related to or apart from weight loss that might also underlie body weight loss‐related improvements in patients with migraine [63].

## FUTURE PERSPECTIVES

Our review provides an overview of the evidence of BT in migraine (Table 5). Our analysis emphasizes the idea that BT impact could be clearly beneficial in migraine management and that it needs to be more extended and should be further analyzed for better defining its use. Its effect would be gradual but durable, because patients are actively involved, and this helps to facilitate awareness and better coping mechanisms. Although we already know some aspects of physiological changes achieved with BT, a better understanding of why BT leads to improved outcomes, the mechanisms of BT that produce these changes in outcomes, and the extent to which combined treatments could lead to better overall outcomes is necessary to provide significant advances in patient care.

**TABLE 5:** Overview of evidence of BT in migraine (based on text information).

Type of behavior therapy	Analyzed aspects	Evidence level (class)
Biofeedback		
To make unconscious or involuntary body processes perceptible to the senses to control them by consciousness	Peripheral skin temperature biofeedback, blood–volume–pulse, and electromyography feedback	I
	Frontal electromyographic biofeedback (combined with pharmacotherapy) to reduce analgesic drug intake	III
	Quantitative electroencephalogram procedure, based on neurofeedback, to reduce migraine frequency	III
	Control of the reflex response and habituation to painful trigeminal stimulation	II
	Reduction in headache frequency (combined with pharmacotherapy)	II
	Decrease in duration of headache	III
Relaxation training		
A systematic procedure for achieving a physical state of relaxation and subsequent mental quietness	The progressive muscle relaxation training reduces migraine frequency and duration	II
	In workplace relaxation exercise programs, reduction in muscle tenderness and decrease in drug intake and lost time at work has been seen	II
	When adding other therapies, such as CBT, a positive result can be observed	III
	Adding CBT seems a good option after withdrawal from medication overuse in patients with chronic migraine	II
	In childhood: Progressive muscle relaxation exercises induce a reduction of >50% of headache days in 50% but did not show differences compared to transcendental meditation and hypnotherapy Music therapy could be an option, although recent studies showed that it is not superior to placebo	II III
Intervention based on mindfulness		
Evidence‐based mind–body intervention that teaches principles of introspection and its application to daily life situations, helping patients to handle illness and stress	Useful for migraine patient's well‐being at emotional and cognitive level	IV
	Improvements in psychological symptoms, pain self‐efficacy and sensory pain perception	III
	MF‐based stress reduction intervention had a beneficial effect on headache duration, disability and self‐efficacy	III
	ACT: A type of behavior therapy based on six core principles: acceptance, cognitive diffusion, contact with the present, self‐in‐context, values, and committed action ACT plus educational workshop exhibited significant improvements in headache frequency, headache severity, medication use, and headache‐related disability	III
Cognitive behavioral therapy		
Based on cognitive and behavioral aspects of migraine, teaching ways to manage mental health‐related factors associated with migraine such as anxiety, depression, sleep disorders, and pain, among others	High rate of subjective improvement of pain and better headache coping using pain‐coping strategies; decreases headache intensity, frequency, and catastrophizing, altering pain‐related cognition of headache patients	II
	Improvement in insomnia, depression, anxiety, and quality of life, producing changes in headache	III
	Patients at risk of medication overuse could benefit from this therapeutic management, especially when combining with bibliotherapy	III
	In childhood, with amitriptyline, improved headache frequency and migraine‐related disability compared to only headache education and amitriptyline	II

Abbreviations: ACT, acceptance and commitment therapy; CBT, cognitive behavior therapy; MF, mindfulness.

Nowadays, migraine therapy is much more developed in pharmacopeia than lifestyle modification, although health professionals know how relevant lifestyle routines are in migraine. The point is that the pharmacopeia and the BT should not be two options, but should be combined as “tools for migraine management,” without either having a leading role, both being adapted to the needs of the person we are trying to help.

Focusing on triggers and life habits broadens the therapeutic option and helps to achieve a much more holistic and personalized management, in this era in which “personalized medicine” is increasingly demanded. In addition to expanding options, BT will reduce the impact of the usual adverse effects of drugs, as well as offer tools to the patient to reduce long‐term costs (in terms of drug and resource costs), and improve, even collaterally, other conditions the patient may suffer that may impact his/her health, related to habitual comorbidities in migraine. Thus, this concludes in acquiring a healthier lifestyle and with that a greater opportunity for well‐being.

However, as we have already seen, this type of treatment has limitations, and time is one of the most common barriers to initiate BT [115], because it is a time‐consuming approach. However, it should be noted that migraine attacks are also time‐consuming; the main difference is that BT time will be of time for well‐being, whereas migraine attacks' time will be bad‐being. Another disadvantage is that active participation and lifestyle modifications are required, which are not always easy to maintain.

That is why BT needs to be considered as an investment and we need to present it as such to the patient, explaining that it might take quite a long time and that it requires patience, perseverance, and constancy, with a high level of motivation. When the main aim is achieved, great impact will be gained, not only in the context of migraine, but also in the way the patient sees and lives their life. In the long term, and once the patient acquires the necessary knowledge to maintain their healthy routine, it will constitute a relatively low‐cost and accessible nonpharmacological intervention in which the patient's needs are the focus of the disease management. For that, patient education and intervention programs should address other health behaviors focusing on stress management, diet, and sleep health, among others.

Therefore, when managing migraine, a holistic approach that include trigger identification and avoidance and lifestyle modifications with pharmacopeia should be the goal. That is why the management may include also trigger modulation and/or drugs and/or BT if necessary. Due to the relevance of this aspect, when given migraine‐preventive recommendations, the patient must be aware of the options but also of the expected outcomes and the duration of time that might be required to obtain benefits.

We can sum up that integrated and flexible treatment combining different approaches may be more effective than drugs alone. BT helps to alleviate pain and to reinforce clinical improvement. With BT, the autonomy and empowerment of the patient is enhanced, focusing on lifestyle modifications, which in many cases lead to an increase of sense of control and self‐efficacy that can reduce the stress of the unpredictable attacks of pain. Also, some BT treatments like MF are trained in groups, which is beneficial in increasing motivation, engagement in treatment, and other benefits deriving from group support [116]. BT seems to approach the emotional and cognitive aspects of pain, and by consequence to have an effect on the perceptive one. This should be the near future of migraine management along with our advances in pharmacopeia.

However, certain limitations must be recognized in interpreting our results. First, our review is not systematic. We confined our analysis to selected articles that we deemed most representative for the topic of interest. Second, some of these scientific studies contain biases and are conducted with small sample sizes. Moreover, as we can see, only one of the analyzed aspects reaches EL I, eight reach EL II, 10 reach level III, and only one achieves EL IV. That said, it is important to highlight the difficulty for nonpharmacological interventions to meet all the criteria for randomized clinical trials to achieve a high EL. It is not possible to respect double blinding, and it is very difficult to find sham interventions; this also biases results. Hence the need for caution regarding the claims made on them. Third, our search strategy was limited to using only PubMed, which may have restricted the breadth of our narrative review. Among the strengths of our study, we examined a significant number of articles, emphasizing the most pertinent research on BT and migraine highlighting the EL of the claim according to the type of study conducted.

## CONCLUSIONS

As a summary, despite the lack of strong evidence, there are several studies from which we could conclude that BT could be a good choice in migraine management, for acute treatment but also as a preventive therapy. It seems useful mainly combined with other options such as pharmacotherapy and especially for motivated patients whose quality of life is affected by modifiable factors. BT could modulate migraine burden by its action on cortical factors and trigeminal activation. Thus, RT seems to decrease the sympathetic nervous system's response to stress, with which migraine pain tolerance increases, also combined with CBT.

CBT, especially used together with pharmacological treatment, can increase pain‐coping strategies for patients at risk of medication overuse and those with insomnia or mental health‐related factors. These are considered the most suitable patient populations for a good response to BT. MF has demonstrated effectiveness in reducing pain‐related distress and in enhancing heart rate variability. It is expected to act primarily on headache‐related disability and secondarily on headache duration and self‐efficacy. Finally, ACT seems suitable to manage stress related to pain onset and the awareness of the pain and its origin. Along these lines, mobile health and electronic health applications may improve BT by improving awareness and adjustment to healthier lifestyle modifications.

Looking for the most suitable patient profile when there is a risk of medication overuse, MF‐CBT among others seems to be a good choice, with similar results compared to pharmacological prophylaxis but without adverse effects. Despite the lack of evidence supporting this type of therapy in children and adolescents, adding BT is postulated to be even more effective than usual care in this age group.

In general, BT shows promising outcomes, although there is a need for future research to strengthen the available evidence. Moreover, as the ELs for these nonpharmacological therapies are low, specific knowledge and training are required for headache health professionals to practice BT and for BT health professionals to treat headache patients.

We conclude that BT is a useful tool with long‐lasting value. Therefore, migraine long‐term treatment should focus on adherence to therapy as well as on medical information and lifestyle modification options. Data from this study encourage the incorporation of nonpharmacological interventions in migraine treatment, and the investigation of these techniques to profile the patient features for which each evaluated technique may be more suitable and thus to obtain a greater return on the investment made while working on it.

## AUTHOR CONTRIBUTIONS

**Ane Mínguez‐Olaondo:** Investigation; formal analysis; writing – original draft; writing – review and editing; project administration; supervision; conceptualization; data curation. **Patricia Alves Días:** Investigation; conceptualization; formal analysis; writing – original draft; data curation. **Estibaliz López de Munáin:** Investigation; formal analysis; writing – original draft; data curation; conceptualization. **Vesselina Grozeva:** Writing – original draft. **Carmen Laspra‐Solís:** Writing – review and editing. **Inés Martín Villalba:** Writing – review and editing; writing – original draft. **Valvanuz García‐Martín:** Writing – review and editing. **Marta Vila‐Pueyo:** Conceptualization; investigation; formal analysis; writing – review and editing. **Myriam Barandiarán:** Writing – review and editing. **Ramon J. Zabalza:** Writing – review and editing. **Ana Bengoetxea:** Conceptualization; investigation; formal analysis; writing – review and editing.

## FUNDING INFORMATION

There were no funders involved in the study design, collection, analysis, interpretation of data, the writing of this article, or the decision to submit it for publication.

## CONFLICT OF INTEREST STATEMENT

The authors declare that the research was conducted in the absence of any commercial or financial relationships that could be construed as a potential conflict of interest.

## Data Availability

Data sharing is not applicable to this article as no new data were created or analyzed in this study.

## ANNEX I. EVIDENCE LEVEL A

Based on those used by the American Academy of Neurology (AAN), the standard definitions of classification of evidence are as follows:

### A.1. CLASS I

Evidence provided by one or more well‐designed, randomized, controlled clinical trials, including overviews (meta‐analyses) of such trials.

### A.2. CLASS II

Evidence provided by a well‐designed, matched‐group cohort study or by a randomized, controlled clinical trial lacking in one design criterion.

### A.3. CLASS III

Evidence provided by other controlled trials such as those with natural history controls or patients serving as their own controls, with independent outcome assessments.

### A.4. CLASS IV

Evidence provided by expert opinion, case series, case reports, or uncontrolled studies. For more detailed guidance, see Appendix 9 in the AAN Clinical Practice Guideline Process Manual, 2004 Edition, available at http://www.aan.com/globals/axon/assets/2535.pdf.
